# Empathy training models and the effects on psychological concerns in paid and unpaid caregivers of older people: A systematic review

**DOI:** 10.1192/j.eurpsy.2023.540

**Published:** 2023-07-19

**Authors:** R. M. D. P. P. Pessoa, M. A. Maximiano-Barreto, A. C. Ottaviani, B. M. Luchesi, M. H. N. Chagas

**Affiliations:** ^1^University of São Paulo, Ribeirão Preto; ^2^University of São Carlos, São Carlos; ^3^Federal University of Mato Grosso do Sul, Três Lagoas, Brazil

## Abstract

**Introduction:**

Empathy training directed at empathic abilities and/or aspects of providing care can be effective at increasing levels of this ability. Moreover, training in different care contexts can minimize the negative impacts of providing care.

**Objectives:**

To identify empathy training models and the effects on psychological concerns in paid and unpaid caregivers of older people.

**Methods:**

A systematic review was conducted. Searches for relevant articles were performed in the Embase, LILACS, PsycInfo, Pubmed, Scopus and Web of Science databases using the following search strategy: *“Empathy AND (Education OR Training OR Intervention) AND Caregiver”.* No restrictions were imposed regarding language or year of publication.

**Results:**

Empathy training for caregivers of older people were performed in six studies, three of which identified a significant increase in empathy levels and consequent reduction in psychological concerns. Empathy training focused on aspects of empathy and/or the caregiver had significant effects on the outcome variables. Moreover, training conducted online, by telephone and/or in person can generate satisfactory results. The other three studies that conducted training with a focus on aspects of dementia and/or old age did not present any effect on the outcome variables.

**Conclusions:**

Empathy training for caregivers of older people can increase levels of this ability, especially in the cognitive domain, as well as diminish psychological concerns caused by the negative impact of providing care.

**Disclosure of Interest:**

None Declared

